# Supplementary material for: Pre-aged terrigenous organic carbon biases ocean ventilation-age reconstructions in the North Atlantic
Source: Nat Commun. 2023 Jun 24;14:3788. doi: 10.1038/s41467-023-39490-6 (PMC10290711; doi:10.1038/s41467-023-39490-6)
Supplement: Supplementary file 1 — Supplementary Information [file 41467_2023_39490_MOESM1_ESM.pdf]

Supplementary Information
For
Pre-aged terrigenous organic carbon biases ocean ventilation-age reconstructions in the North Atlantic

Jingyu Liu[1,2,8], Yipeng Wang[1,2,8], Samuel L. Jaccard[3], Nan Wang[4], Xun Gong[5,6], Nianqiao Fang[7], Rui Bao[1,2*]

[1]Frontiers Science Center for Deep Ocean Multispheres and Earth System, Key Laboratory of Marine Chemistry Theory and Technology, Ministry of Education, Ocean University of China, Qingdao 266100, China

[2]Laboratory for Marine Ecology and Environmental Science, Pilot Qingdao National Laboratory for Marine Science and Technology, Qingdao 266237, China

[3]Institute of Earth Sciences, University of Lausanne, Lausanne CH-1015, Switzerland

[4]Frontiers Science Center for Deep Ocean Multispheres and Earth System, Key Lab of Submarine Geosciences and Prospecting Techniques, Ministry of Education and College of Marine Geosciences, Ocean University of China, Qingdao 266100, China

[5]Institute for Advanced Marine Research, China University of Geosciences, Guangzhou, China

[6]Shandong Provincial Key Laboratory of Computer Networks, Qilu University of Technology (Shandong Academy of Sciences), Jinan, China

[7]School of Ocean Sciences, China University of Geosciences (Beijing), Beijing 100083, People's Republic of China

[8]These authors contributed equally: J.L. and Y.W.

*Corresponding author (R.B. email: baorui@ouc.edu.cn)

Supplementary Table. 1 Sites information mentioned in this study

Site	Regime	Longitude (° E)*	Latitude (° N)*	Water depth (m)	Reference
PS1243	Nordic seas (Arctic Mediterranean)	6.55	69.37	2700	1
RAPiD-10-1P	South Iceland Rise (high-latitude central North Atlantic)	-17.59	62.98	1200	2
RAPiD-15-4P	South Iceland Rise (high-latitude central North Atlantic)	-17.13	62.29	2100	2
RAPiD-17-5P	South Iceland Rise (high-latitude central North Atlantic)	-19.54	61.48	2300	2
IODP U1314	Gardar Drift (high-latitude central North Atlantic)	-27.89	56.36	2820	this study
IODP U1308	Ruddiman’s IRD belt (high-latitude central North Atlantic)	-24.24	49.88	3870	this study
IODP U1302	Orphan Knoll (high-latitude western North Atlantic)	-45.64	50.17	3560	this study
Null	Manning seamount (middle-latitude western North Atlantic)	-60.50	38.00	1710–1890	3
Null	Muir seamount (middle-latitude western North Atlantic)	-62.50	33.50	1380–2440	3
Null	Gregg seamount (middle-latitude western North Atlantic)	-61.00	39.00	1180–1220	3
KNR140 56GGC	Carolina slope (middle-latitude western North Atlantic)	Null	Null	1400	3
KNR140 51GGC	Carolina slope (middle-latitude western North Atlantic)	Null	Null	1790	3
HU72021-3	Scotian Margin (middle-latitude western North Atlantic)	Null	Null	2470	3
KNR140 39GGC	Blake Ridge (middle-latitude western North Atlantic)	Null	Null	2975	3
KNR140 30GGC	Blake Ridge (middle-latitude western North Atlantic)	Null	Null	3433	3
OCE326 14GGC	Laurentian Fan (middle-latitude western North Atlantic)	Null	Null	3525	3
HU73031-7	Laurentian Fan (middle-latitude western North Atlantic)	Null	Null	4055	3
KNR140 12JPC	Blake Ridge (middle-latitude western North Atlantic)	Null	Null	4250	3
OCE326 GGC-5	Bemuda Rise (middle-latitude western North Atlantic)	Null	Null	4600	3
MD99-2334K	Iberian Margin (middle-latitude eastern North Atlantic)	-10.17	37.8	3146	4
SHAK06-5K	Iberian Margin (middle-latitude eastern North Atlantic)	-10.15	37.57	2646	5
Null	Equatorial Atlantic (low-latitude North Atlantic)	-21.31–-44.56	5.62–10.74	973–1162	6
MD07-3076	Eastern flank of the mid-Atlantic ridge (South Atlantic)	-14.21	-44.07	3770	7

* The minus symbols represent the west longitude or south latitude. ‘Null’ indicates no data from the references.

Supplementary Discussion:

The potential influence of hydrodynamic processes and bioturbation

While OC aging driven by hydrodynamic processes has been reported on continental shelves[8,9], the pre-aging extent would be up to ~2,000 yr. However, it is difficult to interpret the extremely high ^{14}C age difference between OC and coeval planktonic foraminifera (~ 4240 yr to 15440 yr) during HS1 considering only hydrodynamic processes. Prior study suggested that HS1 and YD may have been characterized by much weaker hydrodynamic conditions[10,11]. In consequence, compared to co-deposited foraminifera (~ 4240 yr to 15440 yr) during HS1 particularly when considering the negative $\delta^{13}C_{org}$ values at Site U1308 (Fig. 2), the much older sedimentary OC would be more likely to due to enhanced input of pre-aged terrigenous OC to the North Atlantic. We thus consider that such large age offsets (~ 4240 yr to 15440 yr) may mainly arise from the contribution of pre-aged OC remineralization in the subsurface ocean, while OC aging related to hydrodynamical processes may also have played a secondary role in affecting radiocarbon ages.

In addition, bioturbation may affect the vertical OC age distribution in the sediment core[12]. In our case, the largest age difference between OC and planktic foraminifera is reported from Section U1308B-1H-1 (84-86 cm) (i.e. 15.8 ka BP). Prior study has suggested that bioturbation decreased with depth during HS1 at this site[12]. It thus implies that bioturbation would tend to mix younger sediment downwards, which may cause a larger age offset. We thus assume that even though bioturbation may have affected our sediment deposits, our age offset estimates remain conservative if anything, which does not challenge our conclusions.

Supplementary Figures

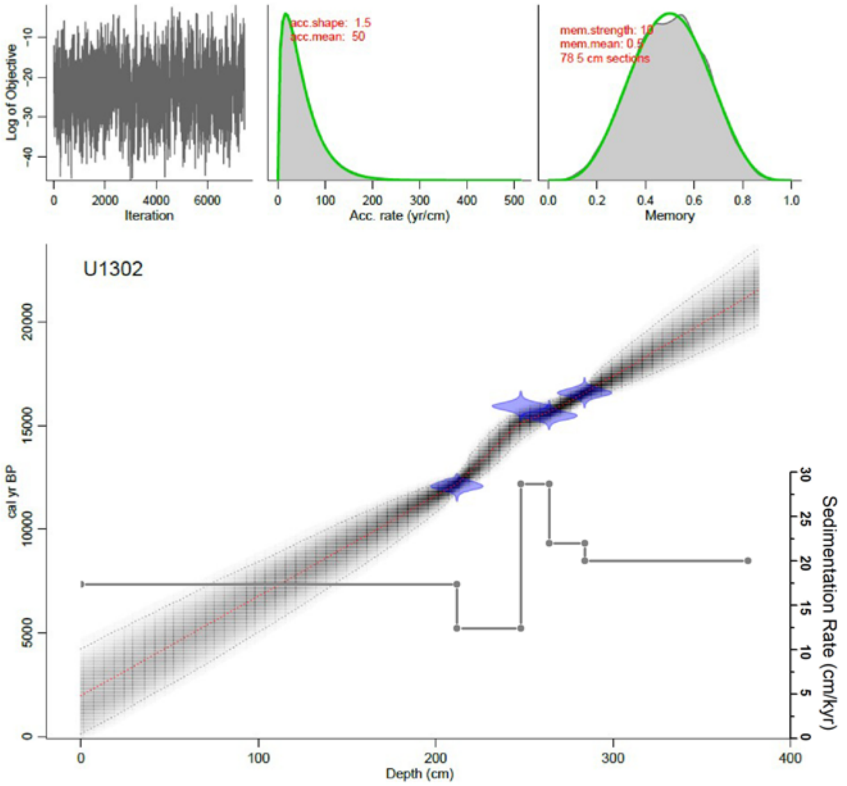

Supplementary Fig. 1 | Age model for site U1302. The age model was constructed using four planktic foraminifera (*N. pachyderma*) ^{14}C dates and the Bacon modelling[13]. Darker grey shading indicates more likely calendar ages; grey stippled lines show 95% confidence intervals; the red curved line shows the single "best" model based on the weighted mean age for each depth. Because ^{14}C data from only four points were used for the last deglaciation, the uncertainty of the age model beyond this time interval increased.

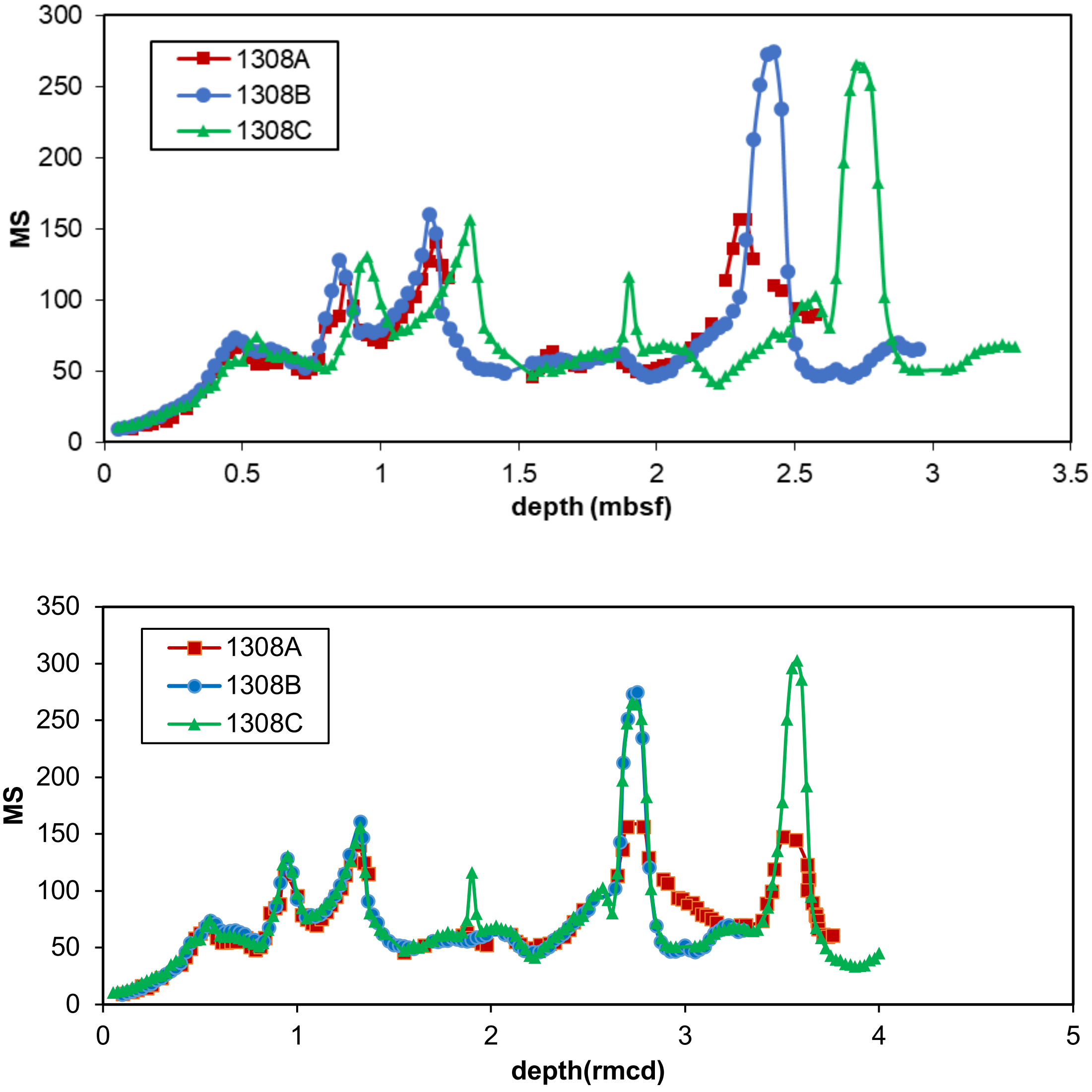

Supplementary Fig. 2 | Mbsf (meters below sea floor) and rmcd (revised meters composite depth) values for site U1308. Core samples (U1308A, U1308B, and U1308C) were used to revise each depth to a consistent composite meter depth by tuning the magnetic susceptibility (MS).

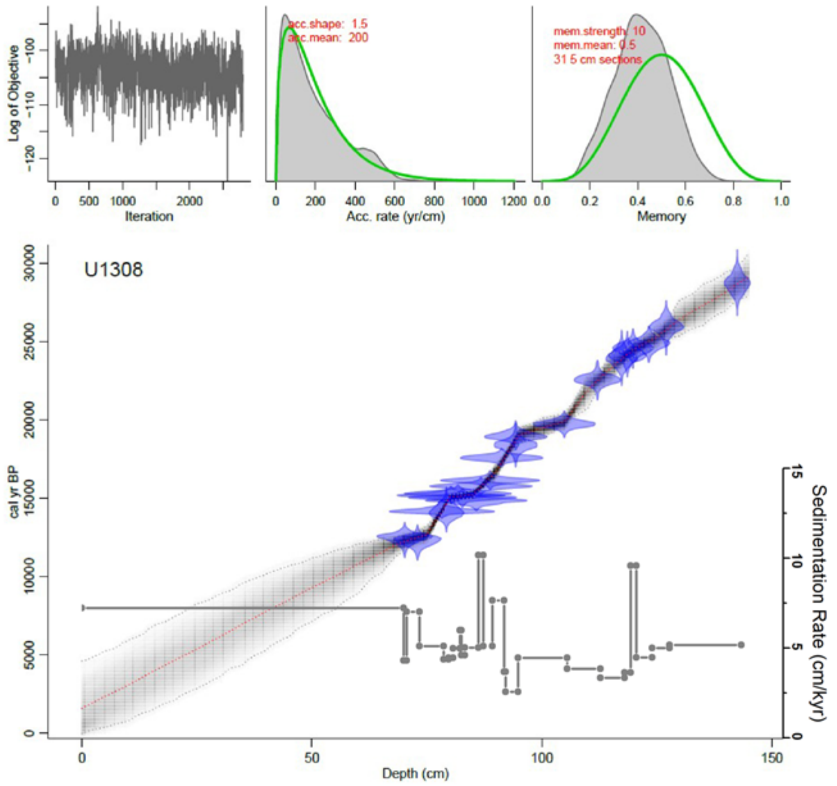

Supplementary Fig. 3 | Age model for site U1308. The age model of site U1308 was built using ^{14}C data from previous studies[14], rmcd values, and the new ^{14}C calibration curve for Marine20[15]. Darker grey shading indicates more likely calendar ages; grey stippled lines show 95% confidence intervals; and the red curve shows the single "best" model based on the weighted mean age for each depth.

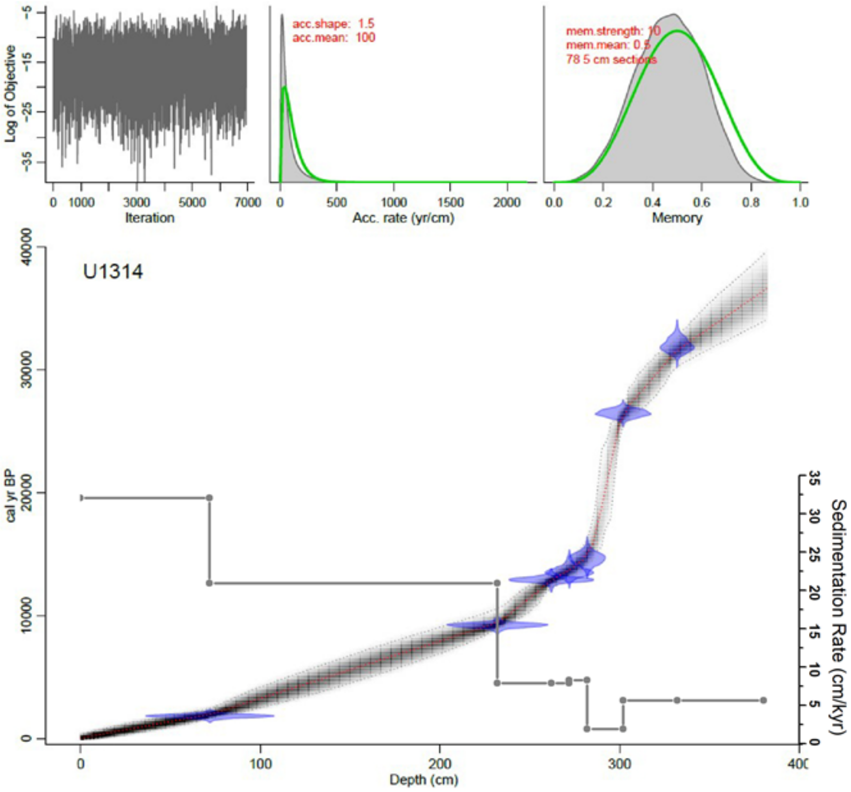

Supplementary Fig. 4 | Age model for site U1314. An age model was constructed using seven planktic foraminifera (*G. bulloides*) ^{14}C dates and Bacon modelling[13]. Darker grey shading indicates more likely calendar ages; grey stippled lines show 95% confidence intervals; and the red curve shows single "best" model based on the weighted mean age for each depth.

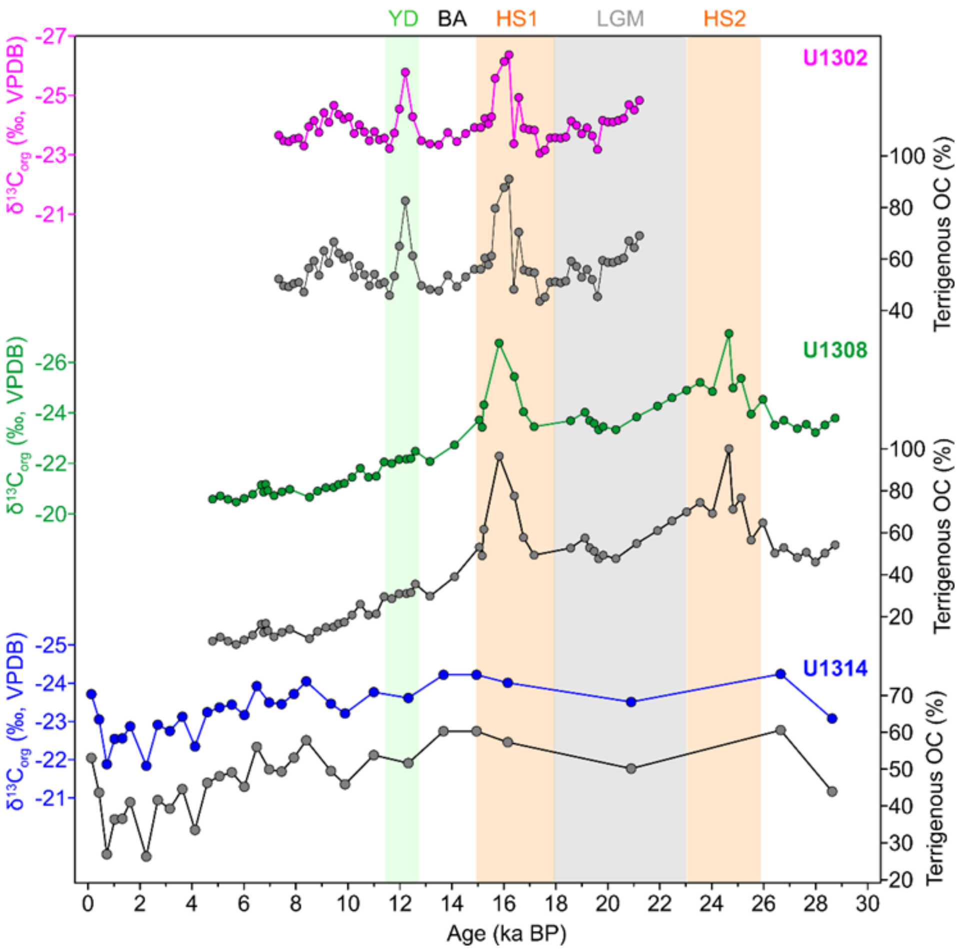

Supplementary Fig. 5 | Percentages of terrigenous OC were calculated using the two-end member model for $\delta^{13}C_{org}$. The end-member values were determined as described in the Methods section. Clearly high terrigenous Organic Carbon (OC) percentages were observed with the terrigenous OC during HS1, even up to >90% at sites U1302 and U1308.

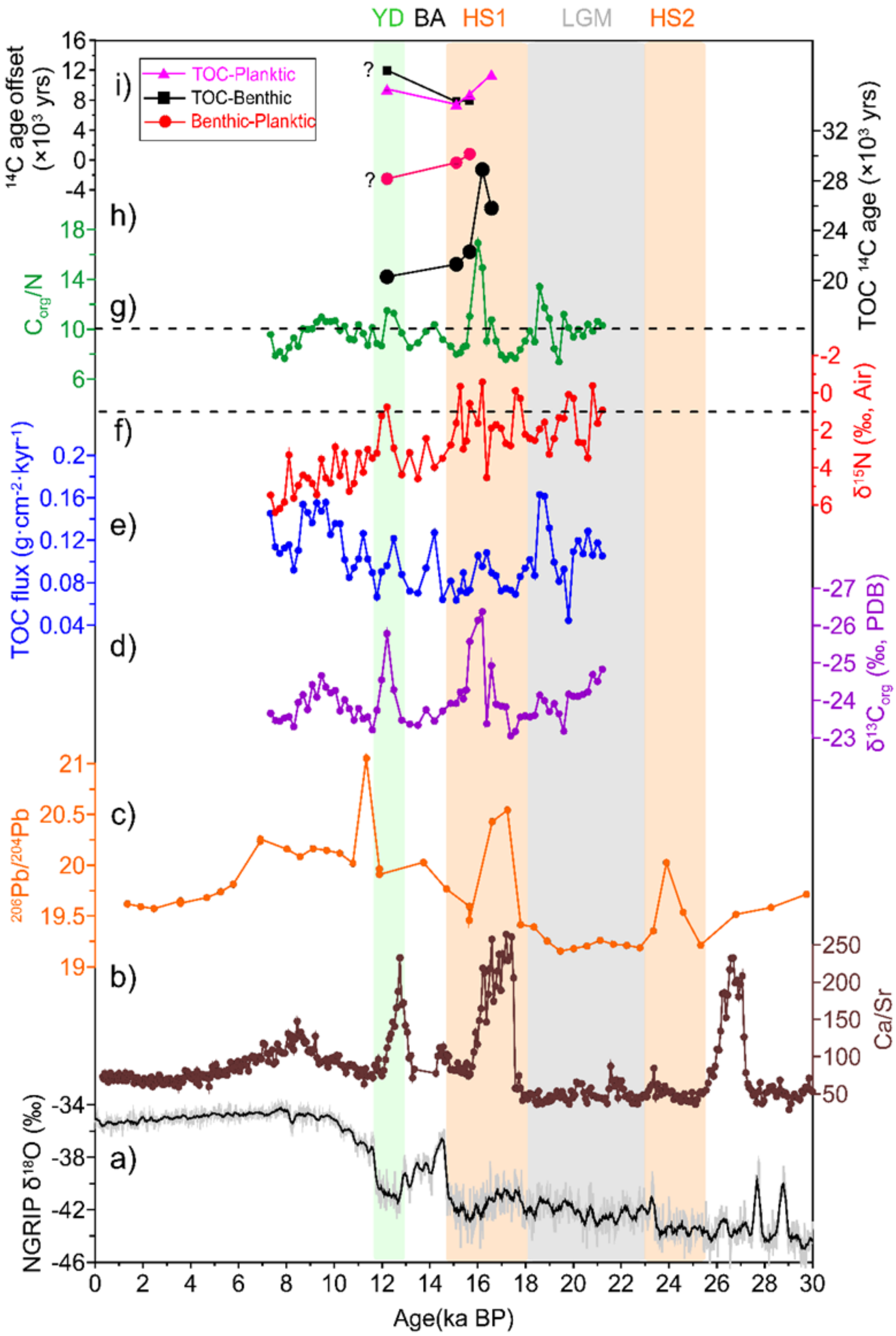

Supplementary Fig. 6 | Variations in multiple proxies at site U1302 since the last glaciation. **a**, The δ^{18}O curve of the Greenland Ice Core from the NGRIP present a referenced chronology[16]; the grey line represents the raw data and the black line represents the data after 10-point smoothing. **b**, Ca/Sr ratio of bulk sediments[17]. **c**, Proxy ^{206}Pb/^{204}Pb ratio to indicate terrigenous input from a previous study[18]. **d**, $\delta^{13}C_{org}$ values of the bulk sediments. **e**, Total Organic Carbon (TOC) fluxes based on the densities from proceedings of the IODP and sediment rates of our new age model. **f – g**, δ^{15}N and C/N values of bulk sediments that indicate terrigenous

inputs; the dashed lines show the boundaries used to identify terrigenous (upper) and marine (lower) OC[19,20]. **h**, ^{14}C ages of TOC. **i**, ^{14}C age offsets between TOC and planktonic foraminifera (purple triangles), TOC and benthic foraminifera (blank squares), and benthic and planktonic foraminifera (red circles). Clear TOC aging during the HS1 and YD period was identified by the offset between the TOC and foraminifer ^{14}C ages. Abnormal ^{14}C values of benthic foraminifera are indicated by question marks. The delay and lead of terrigenous input proxies probably arose from the bias between the different age models referenced here. BA: Bølling–Allerød; HS1: Heinrich Stadial 1; HS2: Heinrich Stadial 2; LGM: Last Glacial Maximum; YD: Younger Dryas

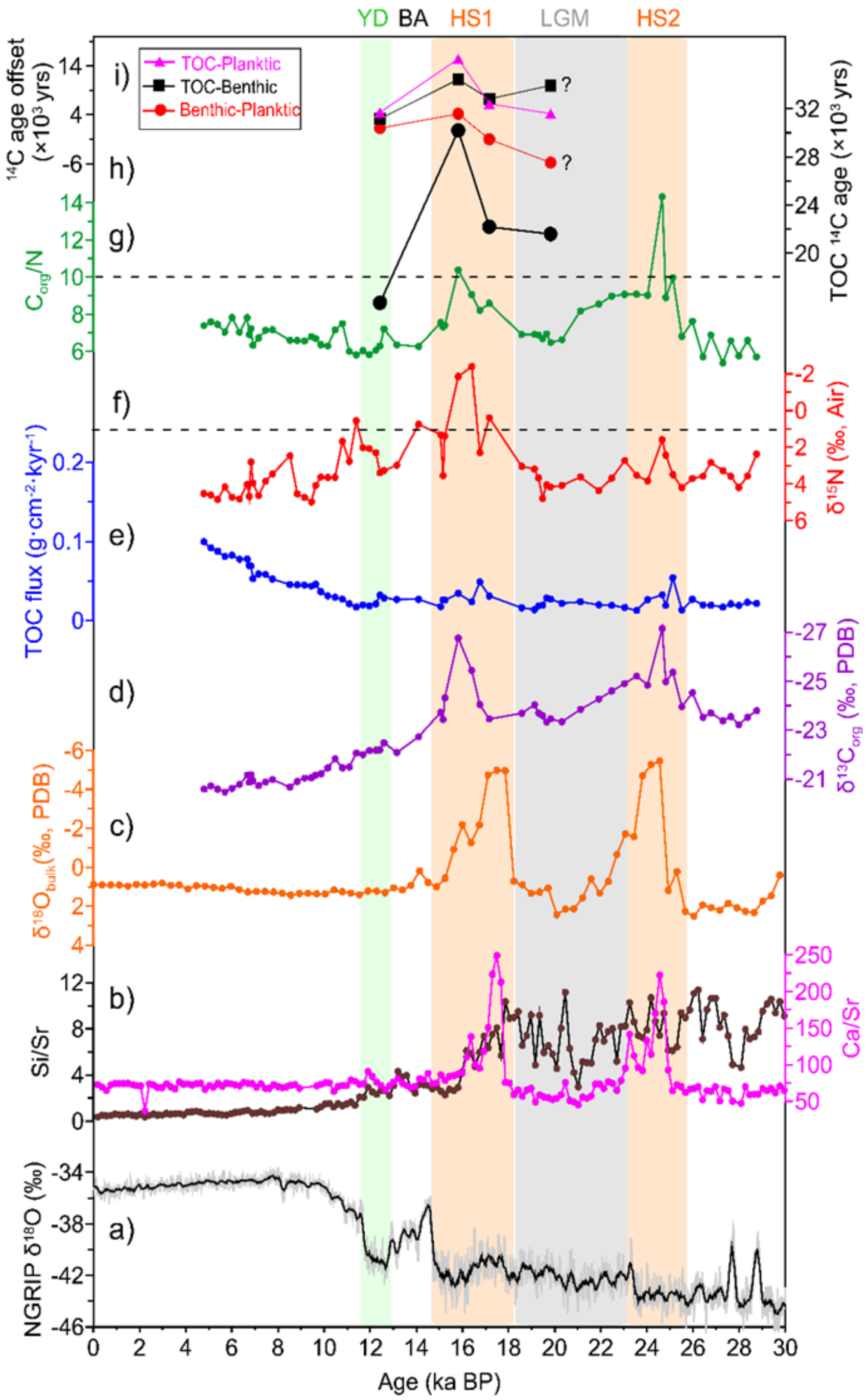

Supplementary Fig. 7 | Variations in multiple proxies at Site U1308 since the last glaciation. **a**, The δ^{18}O curve of the Greenland Ice Core from the NGRIP showed a referenced chronology[16]; the grey line represents the raw data, and the black line represents the data after a 10-point smoothing. **b**, Ca/Sr (purple) and Si/Sr (brown) ratios of bulk sediments[21]. **c**, δ^{18}O data for bulk carbonate determined as indicators of Heinrich events[22]. **d**, $\delta^{13}C_{org}$ values of bulk sediments. **e**, Total Organic Carbon (TOC) flux based on density from the proceedings of the IODP and the sediment rate of our new age model. **f – g**, δ^{15}N and C/N of bulk sediments

that indicated terrigenous inputs; the dashed lines show the boundaries used to identify terrigenous (upper) and marine (lower) OC[19,20]. **h**, ^{14}C age of TOC. **i**, ^{14}C age offsets between TOC and planktonic foraminifera (purple triangles), TOC and benthic foraminifera (blank squares), and benthic and planktonic foraminifera (red circles). Clear aging of OC during HS1 was identified based on the offset between the TOC and foraminifer ^{14}C ages. Abnormal ^{14}C values of benthic foraminifera are indicated with question marks. The delay and lead of terrigenous input proxies probably arose from the bias between the different age models referenced here. BA: Bølling–Allerød; HS1: Heinrich Stadial 1; HS2: Heinrich Stadial 2; LGM: Last Glacial Maximum; YD: Younger Dryas

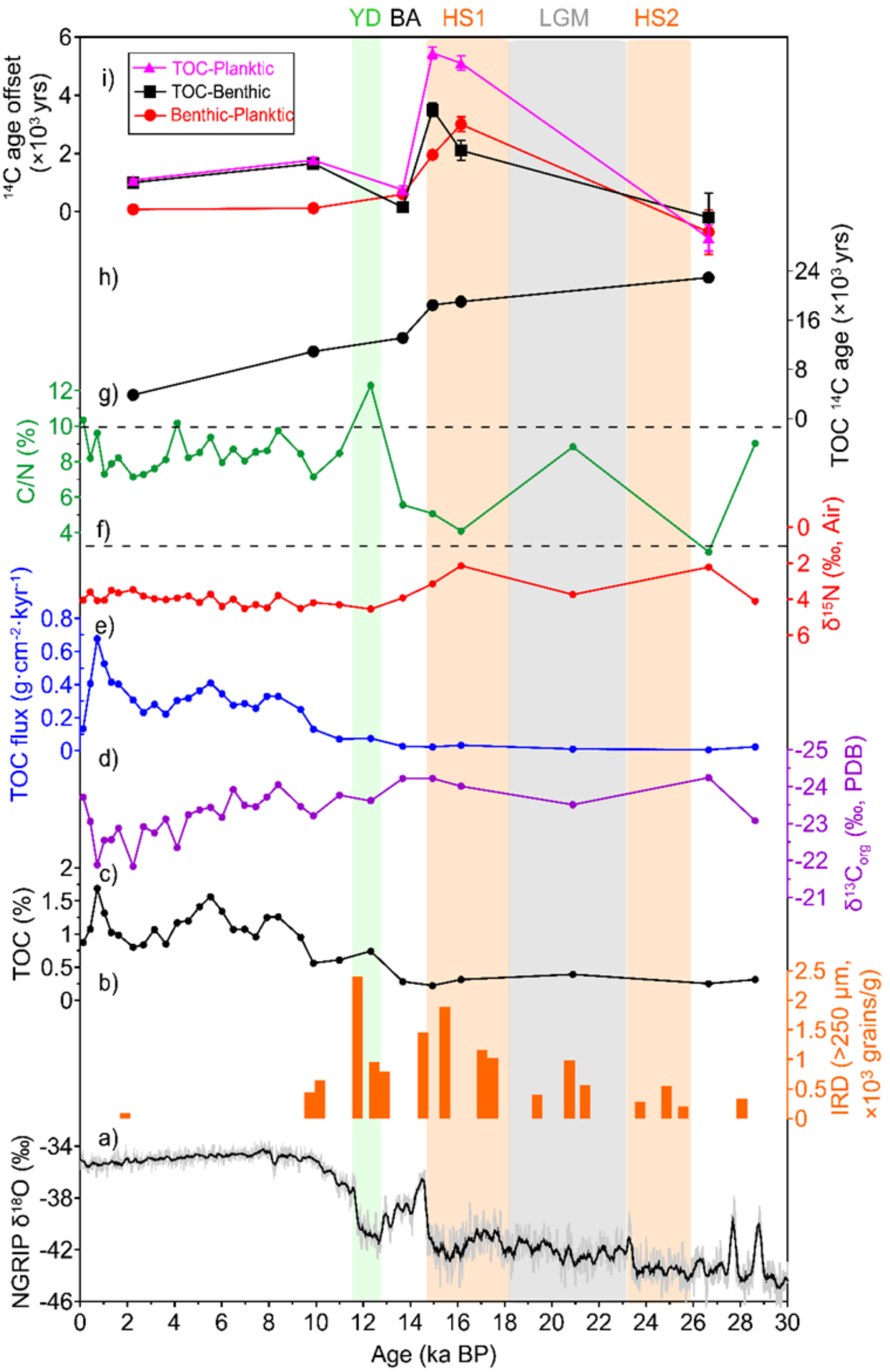

Supplementary Fig. 8 | Variations in multiple proxies at site U1314 since the last glaciation. **a**, The $\delta^{18}O$ curve of the Greenland Ice Core from the NGRIP showed a referenced chronology[16]; the grey line represents the raw data and the black line represents the data after 10-point smoothing. **b**, The IRD data were reported previously[23]. **c**, Total Organic Carbon (TOC) contents. **d**, $\delta^{13}C_{org}$ of the bulk sediments. **e**, TOC flux based on density from the proceedings of the IODP and the sediment rate of our new age model. **f – g**, $\delta^{15}N$ and C/N

of bulk sediments that indicated terrigenous inputs; the dashed lines show boundaries used to identify terrigenous (upper) and marine (lower) OC[19,20]. **h**, ^{14}C age of TOC. **i**, ^{14}C age offsets between TOC and planktonic foraminifera (purple triangles), TOC and benthic foraminifera (blank squares), and benthic and planktonic foraminifera (red circles), with associated 1σ-uncertainties. Clear aging of OC during HS1 was identified based on the offset between the TOC and foraminifer ^{14}C ages. The delay and lead of terrigenous input proxies probably arose from the bias between the different age models referenced here. BA: Bølling–Allerød; HS1: Heinrich Stadial 1; HS2: Heinrich Stadial 2; LGM: Last Glacial Maximum; YD: Younger Dryas

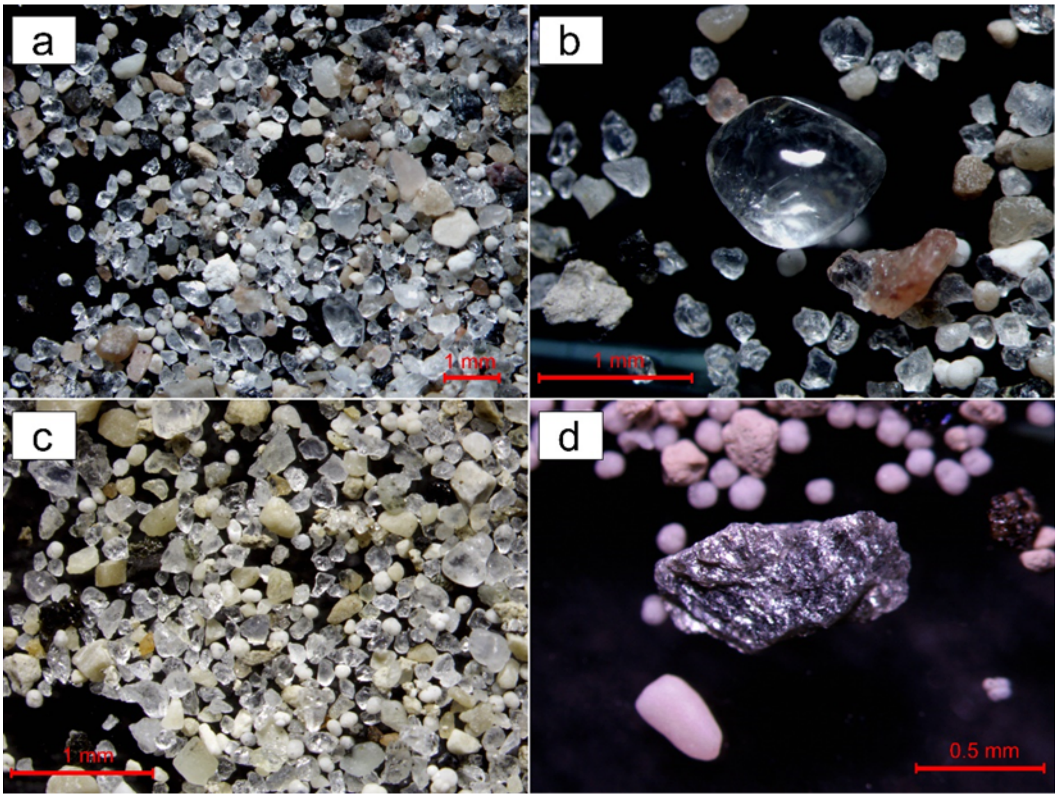

Supplementary Fig. 9 | Microscopic images of sediments with sizes of >150 µm at three sites in the North Atlantic. IRD-enriched layers showed enhanced Organic Carbon (OC) input during HS1. **a**, sample name: U1302D1H2W 134 – 136; **b**, sample name: U1302D1H2W 114 – 116; **c**, sample name: U1308B1H1W 84 – 86; **d**, sample name: U1314B1H2W 132–134.

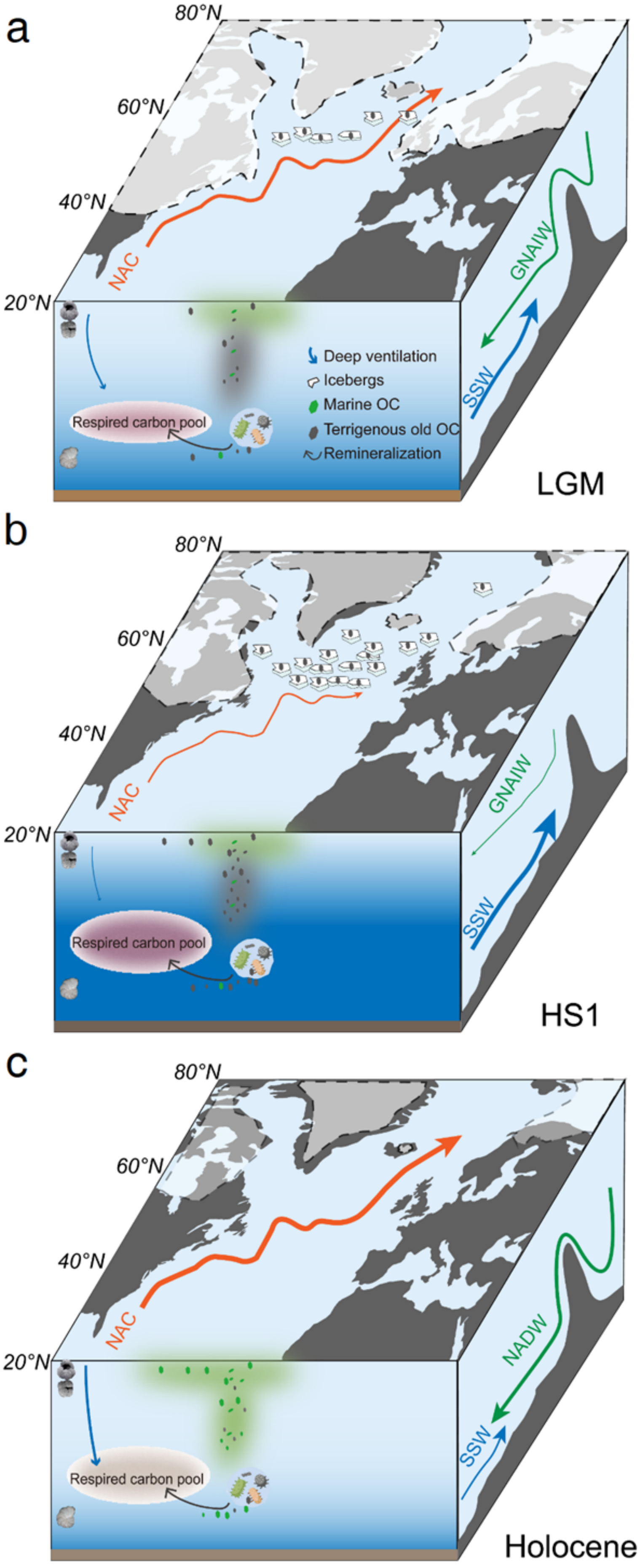
a
80°N
60°N
40°N
20°N
NAC
GNAIW
SSW
Deep ventilation
Icebergs
Marine OC
Terrigenous old OC
Remineralization
Respired carbon pool
LGM
b
80°N
60°N
40°N
20°N
NAC
GNAIW
SSW
Respired carbon pool
HS1
c
80°N
60°N
40°N
20°N
NAC
NADW
SSW
Respired carbon pool
Holocene

Supplementary Fig. 10 | Conceptual model of remineralized old terrigenous Organic Carbon (OC) acting on ventilation. **a – c**, Paleoceanographic changes in the North Atlantic during the LGM, HS1, and Holocene periods. The dashed lines show the ice sheet extent according to previous reports[24-26]. Circulations are indicated by solid lines, where the orange line represents the North Atlantic Current, the green line represents the NADW or the glacial North Atlantic Intermediate Water (GNAIW), and the blue line represents the SSW. The strengths of these circulations are indicated by the sizes of the solid lines. Please note the substantial old terrigenous OC input from icebergs discharging during HS1 was synchronous with poor ocean ventilation. The size and colour of the ellipses reflect the respired carbon pool resulting from OC remineralization. The purple and cream ellipses indicate old and young respired carbon pools, respectively. HS1: Heinrich Stadial 1; LGM: Last Glacial Maximum; SSW: Southern-Sourced Water; NADW: North Atlantic Deep Water.

Supplementary references

1. Thornalley, D. J. R. et al. A warm and poorly ventilated deep Arctic Mediterranean during the last glacial period. *Science* **349**, 706-710 (2015).
2. Thornalley, D. J. R., Barker, S., Broecker, W. S., Elderfield, H. & McCave, I. N. The Deglacial Evolution of North Atlantic Deep Convection. *Science* **331**, 202-205 (2011).
3. Robinson, L. F. et al. Radiocarbon Variability in the western North Atlantic during the last Deglaciation. *Science* **310**, 1469-1473 (2005).
4. Skinner, L. C., Waelbroeck, C., Scrivner, A. E. & Fallon, S. J. Radiocarbon evidence for alternating northern and southern sources of ventilation of the deep Atlantic carbon pool during the last deglaciation. *P. Natl. Acad. Sci. U. S. A.* **111**, 5480-5484 (2014).
5. Ausín, B., Sarnthein, M. & Haghipour, N. Glacial-to-deglacial reservoir and ventilation ages on the southwest Iberian continental margin. *Quat. Sci. Rev.* **255**, 106818 (2021).
6. Chen, T. Y. et al. Persistently well-ventilated intermediate-depth ocean through the last deglaciation. *Nat. Geosci.* **13**, 733-738 (2020).
7. Skinner, L. C., Fallon, S., Waelbroeck, C., Michel, E. & Barker, S. Ventilation of the deep southern ccean and deglacial CO_2 rise. *Science* **328**, 1147-1151 (2010).
8. Ausín, B. et al. Controls on the abundance, provenance and age of organic carbon buried in continental margin sediments. *Earth Planet. Sci. Lett.* **558**, 116759 (2021).
9. Bao, R. et al. Influence of Hydrodynamic Processes on the Fate of Sedimentary Organic Matter on Continental Margins. *Global Biogeochem. Cy.* **32**, 1420-1432 (2018).
10. Mollenhauer, G., McManus, J. F., Wagner, T., McCave, I. N. & Eglinton, T. I. Radiocarbon and 230Th data reveal rapid redistribution and temporal changes in sediment focussing at a North Atlantic drift. *Earth Planet. Sci. Lett.* **301**, 373-381 (2011).
11. Oppo, D. W., Curry, W. B. & McManus, J. F. What do benthic $\delta^{13}C$ and $\delta^{18}O$ data tell us about Atlantic circulation during Heinrich Stadial 1? *Paleoceanography* **30**, 353-368 (2015).
12. Hodell, D. A. et al. Anatomy of Heinrich Layer 1 and its role in the last deglaciation. *Paleoceanography* **32**, 284-303 (2017).

13. Blaauw, M. & Christen, J. A. Flexible paleoclimate age-depth models using an autoregressive gamma process. *Bayesian Anal.* **6**, (2011).

14. Mollenhauer, G. et al. Aging of marine organic matter during cross-shelf lateral transport in the Benguela upwelling system revealed by compound-specific radiocarbon dating. *Geochemistry, Geophys. Geosystems* **8** (2007).

15. Reimer, P. J. et al. The IntCal20 Northern Hemisphere Radiocarbon Age Calibration Curve (0–55 cal kBP). *Radiocarbon* **62**, 725–757 (2020).

16. Svensson, A. *et al.* A 60 000 year Greenland stratigraphic ice core chronology. *Clim. Past* **4**, 47–57 (2008).

17. Grützner, J. & Higgins, S. M. Threshold behavior of millennial scale variability in deep water hydrography inferred from a 1.1 Ma long record of sediment provenance at the southern Gardar Drift. *Paleoceanography* **25**, PA4204 (2010).

18. Crocket, K. C., Vance, D., Foster, G. L., Richards, D. A. & Tranter, M. Continental weathering fluxes during the last glacial/interglacial cycle: insights from the marine sedimentary Pb isotope record at Orphan Knoll, NW Atlantic. *Qua. Sci. Rev.* **38**, 89–99 (2012).

19. Meyers, P. A. Organic geochemical proxies of paleoceanographic, paleolimnologic, and paleoclimatic processes. *Org. Geochem.* **27**, 213–250 (1997).

20. Riethdorf, J.-R. *et al.* Surface nitrate utilization in the Bering sea since 180 kA BP: Insight from sedimentary nitrogen isotopes. *Deep Sea Res. Part II Top. Stud. Oceanogr.* **125–126**, 163–176 (2016).

21. Channell, J. E. T. *et al.* A 750-kyr detrital-layer stratigraphy for the North Atlantic (IODP Sites U1302–U1303, Orphan Knoll, Labrador Sea). *Earth Planet. Sci. Lett.* **317–318**, 218–230 (2012).

22. Hodell, D. A., Channell, J. E. T., Curtis, J. H., Romero, O. E. & Röhl, U. Onset of "Hudson Strait" Heinrich events in the eastern North Atlantic at the end of the middle Pleistocene transition (~640 ka)? *Paleoceanography* **23**, PA4218 (2008).

23. Alvarez Zarikian, C. A., Stepanova, A. Yu. & Grützner, J. Glacial–interglacial variability in deep sea ostracod assemblage composition at IODP Site U1314 in the subpolar North Atlantic. *Mar. Geol.* **258**, 69–87 (2009).

24. Hughes, A. L. C., Gyllencreutz, R., Lohne, Ø. S., Mangerud, J. & Svendsen, J. I. The last Eurasian ice sheets – a chronological database and time-slice reconstruction, DATED-1. *Boreas* **45**, 1–45 (2016).

25. Stokes, C. R., Margold, M., Clark, C. D. & Tarasov, L. Ice stream activity scaled to ice sheet volume during Laurentide Ice Sheet deglaciation. *Nature* **530**, 322–326 (2016).

26. Andrews, J. T. & Voelker, A. H. L. "Heinrich events" (& sediments): A history of terminology and recommendations for future usage. *Qua. Sci. Rev.* **187**, 31–40 (2018).
