# Supplementary material for: Pre-aged terrigenous organic carbon biases ocean ventilation-age reconstructions in the North Atlantic
Source: Nat Commun. 2023 Jun 24;14:3788. doi: 10.1038/s41467-023-39490-6 (PMC10290711; doi:10.1038/s41467-023-39490-6)
Supplement: Supplementary file 3 — Description of Additional Supplementary Files [file 41467_2023_39490_MOESM3_ESM.pdf]

Description of Additional Supplementary Files:

Supplementary Data 1: ^{14}C data measured in this study

Supplementary Data 2: ^{14}C ages and offsets to evaluate the ocean ventilation

Supplementary Data 3: Organic geochemistry proxies of Site U1302

Supplementary Data 4: Organic geochemistry proxies of Site U1308

Supplementary Data 5: Organic geochemistry proxies of Site U1314
